# Cryo-electron microscopic and X-ray crystallographic analysis of the light-driven proton pump proteorhodopsin reveals a pentameric assembly

**DOI:** 10.1016/j.yjsbx.2020.100024

**Published:** 2020-03-08

**Authors:** Stephan Hirschi, David Kalbermatter, Zöhre Ucurum, Dimitrios Fotiadis

**Affiliations:** Institute of Biochemistry and Molecular Medicine, University of Bern, Bühlstrasse 28, 3012 Bern, Switzerland

**Keywords:** Cryo-electron microscopy, Membrane protein, Protein chromatography, Proteorhodopsin, Proton pump, X-ray crystallography, BR, bacteriorhodopsin, BPR, blue-light absorbing proteorhodopsin, BN-PAGE, blue native-polyacrylamide gel electrophoresis, cryo-EM, cryo-electron microscopy, GPR, green-light absorbing proteorhodopsin, IEC, ion exchange chromatography, MW, molecular weight, SDS-PAGE, sodium dodecyl sulphate-polyacrylamide gel electrophoresis, SEC, size exclusion chromatography

## Abstract

The green-light absorbing proteorhodopsin (GPR) is the prototype of bacterial light-driven proton pumps. It has been the focus of continuous research since its discovery 20 years ago and has sparked the development and application of various biophysical techniques. However, a certain controversy and ambiguity about the oligomeric assembly of GPR still remains. We present here the first tag-free purification of pentameric GPR. The combination of ion exchange and size exclusion chromatography yields homogeneous and highly pure untagged pentamers from GPR overexpressing *Escherichia coli*. The presented purification procedure provides native-like protein and excludes the need for affinity purification tags. Importantly, three-dimensional protein crystals of GPR were successfully grown and analyzed by X-ray crystallography. These results together with data from single particle cryo-electron microscopy provide direct evidence for the pentameric stoichiometry of purified GPR.

## Highlights

• Affinity tag-free isolation of proteorhodopsin (PR) by ion exchange and size exclusion chromatography.
• Biochemical and computational analysis indicate a single, pentameric PR population.
• Highly pure and homogeneous PR enables growth of 3D crystals.
• X-ray crystallography and cryo-electron microscopy reveal pentameric assembly of PR.

## Graphical abstract

**Figure:**
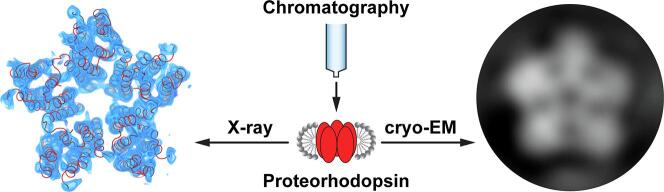

## 1 Introduction

The green-light absorbing proteorhodopsin (GPR), a light-driven proton pump, was discovered 20 years ago in a γ-proteobacterium as the first bacterial rhodopsin (Béjà et al., 2000). A large variety of rhodopsin-based phototrophic systems exist in microorganisms making them highly abundant on earth (Finkel et al., 2013). Within the rhodopsin family, the proteorhodopsins are the most widespread and manifold (Bamann et al., 2014). GPR is the prototype of bacterial rhodopsins and shares ~28% amino acid sequence identity and similar topology (e.g., same number of predicted transmembrane segments) with its well-described archaeal homolog bacteriorhodopsin (BR) (Bamann et al., 2014, Béjà et al., 2001). Both are characterized by a seven transmembrane α-helical structure with an all-*trans* retinal covalently linked via a Schiff base to a conserved lysine residue. Upon absorption of a photon, BR and GPR catalyse the transport of a proton across the cell membrane (Béjà et al., 2001). The photocycle of GPR includes intermediates similar to BR that were termed K (after retinal photoisomerization), M (after deprotonation of the Schiff base), N (after reprotonation of the Schiff base) and O (late intermediate) (Váró et al., 2003). Whereas at alkaline pH BR and GPR share similar functional characteristics, the direction of proton pumping in GPR is inverted at acidic pH (Friedrich et al., 2002). Furthermore, proteorhodopsins exhibit spectral tuning to optimize the absorption of light in phototrophic microorganisms at different depths in the sea. The resulting absorption maximum, e.g., ~525 nm for green-light and ~490 nm for blue-light (BPR) absorbing proteorhodopsins, is mainly determined by an amino acid substitution at residue 105 in GPR and BPR (Ozaki et al., 2014). Microorganisms containing BPR are more prevalent in deeper waters where the blue light can still penetrate, while most of the visible spectrum is already filtered out (Man et al., 2003). Another interesting functional feature of GPR is the unusually high pK_a_ of 7.5 for the primary proton acceptor D97, which results mainly from its interaction with H75 (Hempelmann et al., 2011). The discovery and characterization of a variety of new microbial rhodopsins (Gushchin et al., 2013, Inoue et al., 2013) have not only deepened the understanding of their molecular working mechanisms but have initiated the development of various spectroscopic and electrophysiological techniques (Bamann et al., 2014).

Investigation of the oligomeric state of GPR by atomic force microscopy using reconstituted GPR demonstrated the existence of pentamers and hexamers in proteoliposomes (Klyszejko et al., 2008, Shibata et al., 2018). The same oligomeric states were observed by X-ray crystallography of two BPR versions, with *HOT75*BPR forming pentamers and *Med12*BPR hexamers (Ran et al., 2013). Diverging observations using different analytical methods demonstrated the presence of predominantly pentamers or hexamers for GPR purified in the detergent n-dodecyl-β-D-maltopyranoside (DDM) (Edwards et al., 2014, Hoffmann et al., 2010, Hussain et al., 2015, Maciejko et al., 2015, Stone et al., 2013). One study associated the pentameric state of GPR with a specific cross-protomer salt bridge between residues R51 on one and D52 on the adjacent protomer (Maciejko et al., 2015). Disruption of this interaction by point mutations (R51A or D52N) favoured the formation of the hexameric over the pentameric assembly but produced significant amounts of monomers and dimers (Maciejko et al., 2015). It should be noted that different oligomeric states (e.g., monomers, pentamers and hexamers) were observed by analyzing membranes from GPR overexpressing *E. coli* cells that had been treated with a crosslinker (Hussain et al., 2015). This suggests that these states are not an artifact of detergent solubilization or reconstitution into synthetic lipid bilayers. However, all of the above mentioned studies were performed with GPR versions containing a His-tag, which has been shown to potentially cause artificial protein oligomerization (Majorek et al., 2014, Mohanty and Wiener, 2004, Wu and Filutowicz, 1999). Furthermore, the oligomeric state might vary in the expression host and the original organism, because of different lipid bilayer composition. Currently, the cause and effect relationship between the preparation of GPR samples and the stoichiometry of the resulting assembly seems to remain unclear. While there are varying reports on the stoichiometry of the oligomeric assembly, the oligomerization of GPR was shown to be functionally relevant (Hussain et al., 2015). Detergent-solubilized monomeric GPR exhibits a significantly higher pK_a_ for the primary proton acceptor D97 than the oligomer (Hussain et al., 2015). Furthermore, these two forms show different absorption properties and dynamics of the photocycle intermediates. Specifically, a five-fold slower conformational change during the decay of the M state was observed for the oligomer compared to the monomer (Hussain et al., 2015).

Here we present a purification procedure for pentameric GPR that was heterologously expressed in *E. coli* without affinity purification tag and yielded homogeneous and highly pure native-like protein. It was possible to isolate pentameric GPR by combining ion exchange chromatography (IEC) and size exclusion chromatography (SEC). Furthermore, analysis by blue native-polyacrylamide gel electrophoresis (BN-PAGE) indicated the presence of only one oligomeric form. This is an important improvement to previous purifications that yielded either a mixture of pentameric and hexameric GPR, lacked definite proof of the stoichiometry or relied on the use of affinity purification tags (Edwards et al., 2014, Hoffmann et al., 2010, Hussain et al., 2015, Maciejko et al., 2015, Shibata et al., 2018, Stone et al., 2013). The molecular weight (MW) of the ternary protein-detergent-lipid complex determined by SEC and BN-PAGE was used to estimate the stoichiometry of the oligomer in conjunction with molecular simulations. Importantly, we managed to grow three-dimensional protein crystals from purified pentameric GPR. Crystals diffracted below 5 Å resolution, allowing the first structural analysis of GPR by X-ray crystallography. The corresponding electron density revealed the pentameric assembly of the GPR oligomer as well as the heptahelical structure of the individual protomers similar to BPR. Furthermore, the pentameric state of purified GPR was confirmed in solution by single particle cryo-electron microscopy (cryo-EM). The homogeneous and tag-free purification of native-like pentameric GPR paves the way for subsequent functional and structural studies without interference from mixed oligomeric populations.

## 2 Results

### 2.1 Tag-free purification of pentameric GPR

**Fig. 1:**
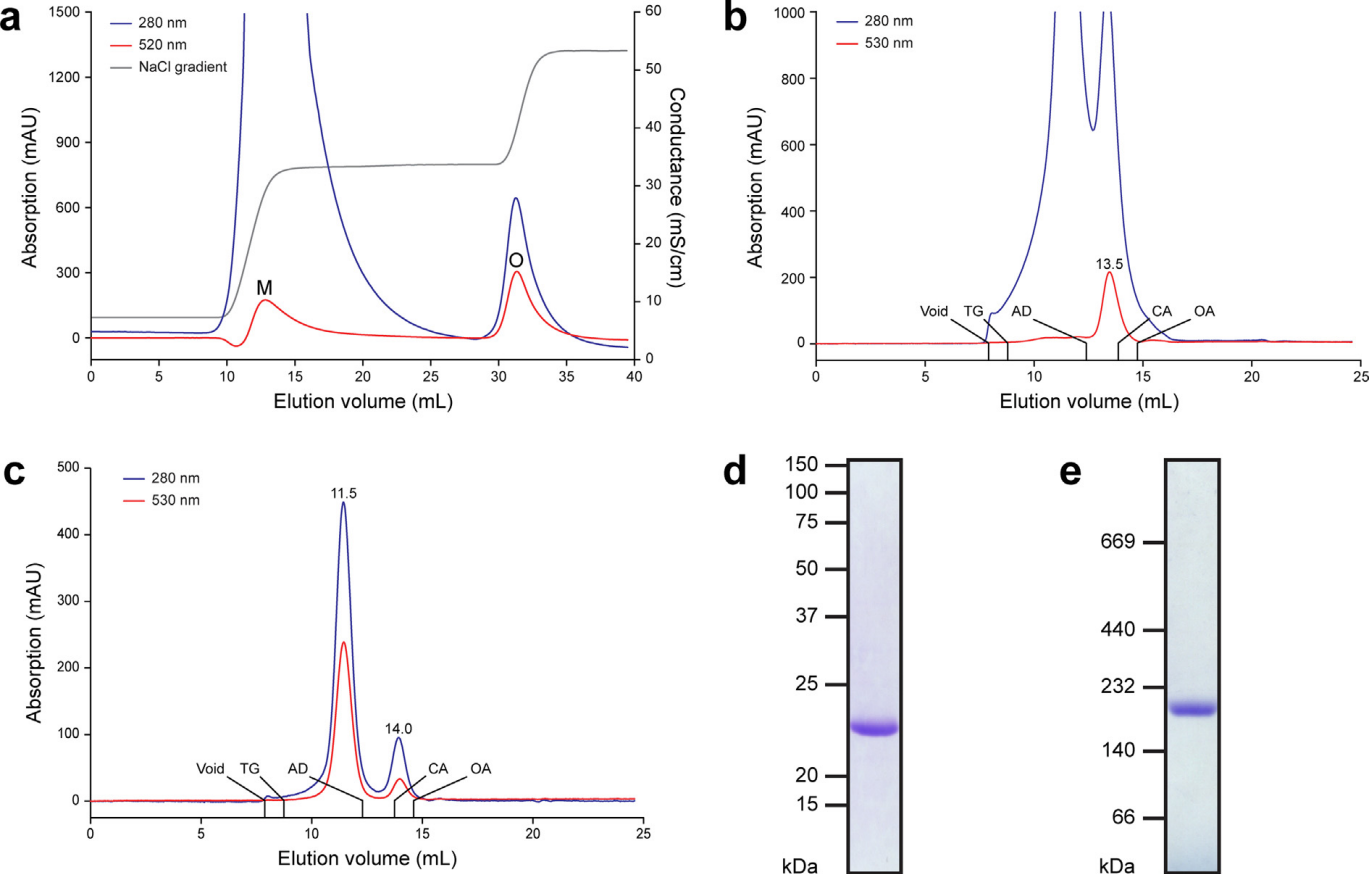
Chromatographic purification of GPR. (a) Anion exchange chromatography elution profile of GPR eluted from Q Sepharose with a NaCl gradient (grey). Absorption of the eluate is shown at 280 nm (blue) and 520 nm (red), revealing two GPR populations (labelled M and O). (b) Size exclusion chromatography elution profile of GPR containing fraction (M) on a Superdex 200 10/300 GL SEC column. (c) Size exclusion chromatography elution profile of GPR containing fraction (O) on a Superdex 200 10/300 GL SEC column. Indicated are the retention volumes for GPR peaks (absorption at 530 nm), void and standard proteins that include thyroglobulin (TG, 669 kDa), aldolase (AD, 158 kDa), conalbumin (CA, 75 kDa) and ovalbumin (OA, 43 kDa). The GPR oligomer purified in Cymal-5 runs as a single band at 23 kDa on a 16% SDS-polyacrylamide gel (d) and at 193 kDa on a 4–16% Bis-Tris BN-polyacrylamide gel (e). (For interpretation of the references to color in this figure legend, the reader is referred to the web version of this article.)

**Fig. 2:**
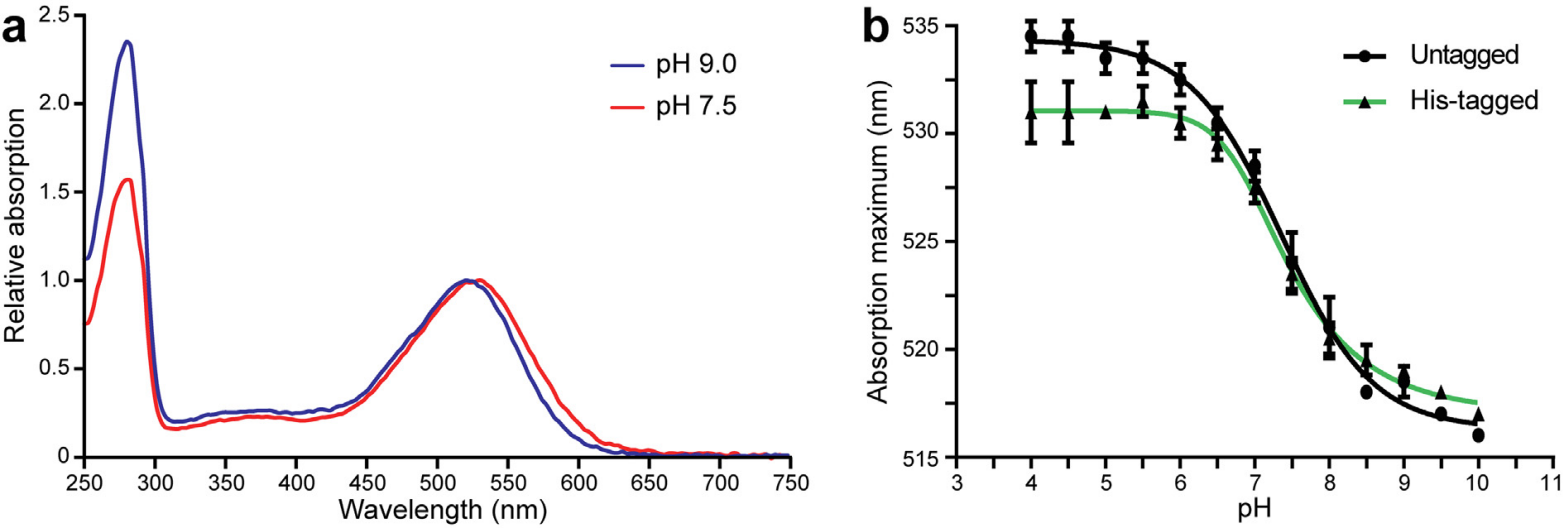
UV–Vis spectroscopic analysis of GPR. (a) UV–Vis spectra of purified GPR at pH 9.0 (after IEC) and pH 7.5 (after SEC) with absorption maxima of the retinal Schiff base at 520 nm and 530 nm, respectively. The lower ratio between maximal absorption at 280 nm and the chromophore peak indicates increased purity after SEC. Spectra were normalized to the respective absorption maxima of the chromophore for better comparison. (b) pH titrations of solubilized *E. coli* membranes containing GPR with or without His-tag. Absorption maxima were plotted against pH and fitted to an asymmetric sigmoidal function using GraphPad Prism, yielding apparent pK_a_ values for the primary proton acceptor of about 7.4. Absorption properties of the two protein versions vary mainly below pH 6, coinciding with the pH range where the His-tag is predominantly protonated.

IEC and SEC revealed that Cymal-5 solubilized membranes from GPR expressing *E. coli* cells contain a mixture of two GPR protein populations (Fig. 1 and Supplementary Fig. 1). They eluted from Q Sepharose resin under different ionic strengths using a NaCl gradient (Fig. 1a). Two main GPR peaks (absorption at 520 nm) were observed beside the remaining membrane protein components (absorption only at 280 nm). The first population of GPR (labelled M in Fig. 1a) eluted immediately after increasing the NaCl concentration above 50 mM whereas the second population (labelled O in Fig. 1a) eluted shortly after 275 mM NaCl. Both peak fractions absorbing at 520 nm were collected and analyzed by SEC (Fig. 1b and c). The first GPR population (M) contained mostly co-purified contaminants as well as GPR eluting at ~13.5 mL (Fig. 1b). The second population (O) consisted of a minor GPR fraction eluting comparable to the first population at ~14 mL and a major GPR fraction eluting at a significantly lower elution volume, i.e., at ~11.5 mL (Fig. 1c). The size of the ternary protein-lipid-detergent complex can be estimated based on the elution volumes of known MW marker proteins (Ilgü et al., 2014). The calculated MWs of the two GPR populations from SEC were 204 kDa (elution volume ~11.5 mL; Fig. 1c), and either 63 kDa (elution volume ~14 mL; Fig. 1c) or 80 kDa (elution volume ~13.5 mL; Fig. 1b). Based on these MWs, the GPR population eluting at lower elution volume corresponds to a higher oligomeric form of GPR. The lower MW population was assigned to monomeric GPR based on comparison with the SEC profile of monomeric LacY in Cymal-5 (elution volume ~13 mL) (Abramson et al., 2003, Ilgü et al., 2014). Furthermore, previous analysis of GPR by BN-PAGE also reported a very similar MW for the monomer (i.e., slightly less than 66 kDa), whereas a dimer was shown to be significantly larger (i.e., slightly less than 146 kDa) (Maciejko et al., 2015). Sodium dodecyl sulphate-polyacrylamide gel electrophoresis (SDS-PAGE) of the purified GPR oligomer showed a single band at 23 kDa, which corresponds to the MW of monomeric GPR (Fig. 1d). No contaminants were visible in the SDS-polyacrylamide gel, demonstrating the high purity of isolated GPR. During purification the protein purity can be followed by comparing the ratio of the absorption at 280 nm and the chromophore peak in the UV–Vis absorption spectrum. The lower ratio after SEC compared to before (i.e., after IEC) indicates increased purity (Fig. 2a). The MW of the oligomeric GPR complex purified in Cymal-5 was confirmed by BN-PAGE (Fig. 1e) yielding 193 kDa, in line with the MW obtained from SEC. Importantly, a single protein band was observed on BN-PAGE indicating the presence of only one oligomeric GPR form. In addition, we directly analyzed membranes of GPR expressing *E. coli* solubilized under various conditions by SEC to detect potential GPR populations of different oligomeric states. Similar to the findings for the GPR homolog Gloeobacter rhodopsin (Morizumi et al., 2019), we found that GPR exhibits a pH-dependent transition from oligomer to monomer when exposed to acidic conditions (Supplementary Fig. 1a). GPR solubilized in DDM forms mainly oligomers of comparable size to Cymal-5, excluding a detergent specific oligomeric state (Supplementary Fig. 1b). Comparison by BN-PAGE of untagged and His-tagged GPR containing *E. coli* membranes solubilized in DDM indicated no difference in oligomeric state regardless of the presence of a His-tag (Supplementary Fig. 2).

**Fig. 3:**
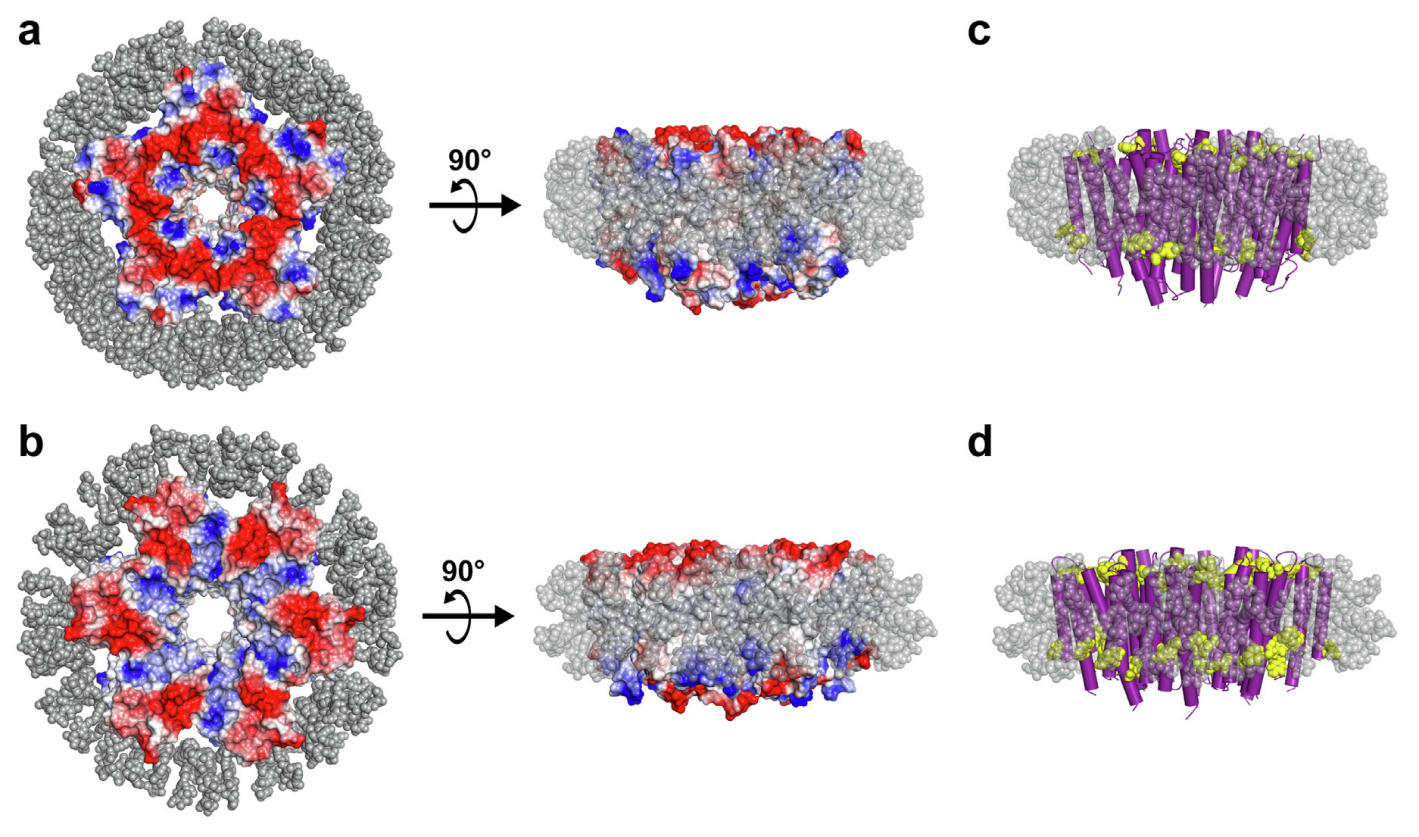
Models of BPR-Cymal-5 protein-micelle complexes. Top view and side view of (a) pentameric BPR (PDB ID: 4KLY) modelled with 155 Cymal-5 molecules (grey) and (b) hexameric BPR (PDB ID: 4JQ6) with 103 Cymal-5 molecules. Protein electrostatics are displayed as negative (red), positive (blue) and neutral (white) potentials. Side view of the pentamer (c) and hexamer (d) detergent complex with BPR transmembrane α -helices depicted as cylinders (purple) and highlighted (yellow) aromatic side chains (Trp, Tyr and His) close to the lipid-water interface. Detergent micelles are displayed transparently in all side views for better clarity. The displayed structural information was prepared using PyMOL (https://pymol.org). (For interpretation of the references to color in this figure legend, the reader is referred to the web version of this article.)

Using the MWs of the GPR oligomer-lipid-detergent complex determined by SEC (204 kDa) or BN-PAGE (193 kDa) the approximate size of the detergent micelle can be calculated. The theoretical MW of one GPR monomer is 25.5 kDa, as calculated from the amino acid sequence. The remaining MW of 76.5 kDa (or 65.5 kDa from BN-PAGE) for a potential pentameric, or 51 kDa (or 40 kDa from BN-PAGE) in case of a potential hexameric assembly, can be attributed to the detergent micelle and co-purified lipids. In an approximation, i.e., neglecting potential lipids, a complex consisting only of protein and detergent was modelled for the BPR pentamer and hexamer (Fig. 3). The higher MW calculated from SEC was used to not underestimate the detergent content. The resulting pentamer complex contained 155 and the hexamer 103 Cymal-5 molecules. The hydrophobic surfaces of the protein (depicted in white) are not completely protected by the detergent micelle surrounding the hexamer while in the pentamer they are almost entirely covered. Insufficient detergent content in the hexamer suggests that the purified GPR oligomer is most likely a pentamer. Additionally, the boundaries confined by the “aromatic belt” (a ring of aromatic residues commonly found at the lipid-water interface of membrane proteins (Schabert et al., 1995)) matches the borders of the detergent micelle (Fig. 3c and d) similar to experimental data from neutron diffraction studies (Pebay-Peyroula et al., 1995). This is an indication that the protein-detergent complexes were correctly modelled.

To compare the functional characteristics of GPR with and without His-tag, pH titrations were performed to assess the absorption properties of the Cymal-5 solubilized proteins (Fig. 2b). From the relation of the absorption maximum of the retinal Schiff base and the pH, an apparent pK_a_ of the primary proton donor can be calculated (Ozaki et al., 2014). Both titrations of GPR, with and without His-tag, yielded pK_a_ values of about 7.4, suggesting that the presence of a decahistidine-tag does not significantly affect the functional features of GPR. However, it is interesting to note that at pH 6.0 and lower, the absorption maximum of the His-tagged protein is slightly blue-shifted compared to GPR without His-tag.

### 2.2 Structural analysis of GPR by X-ray crystallography and cryo-EM

**Fig. 4:**
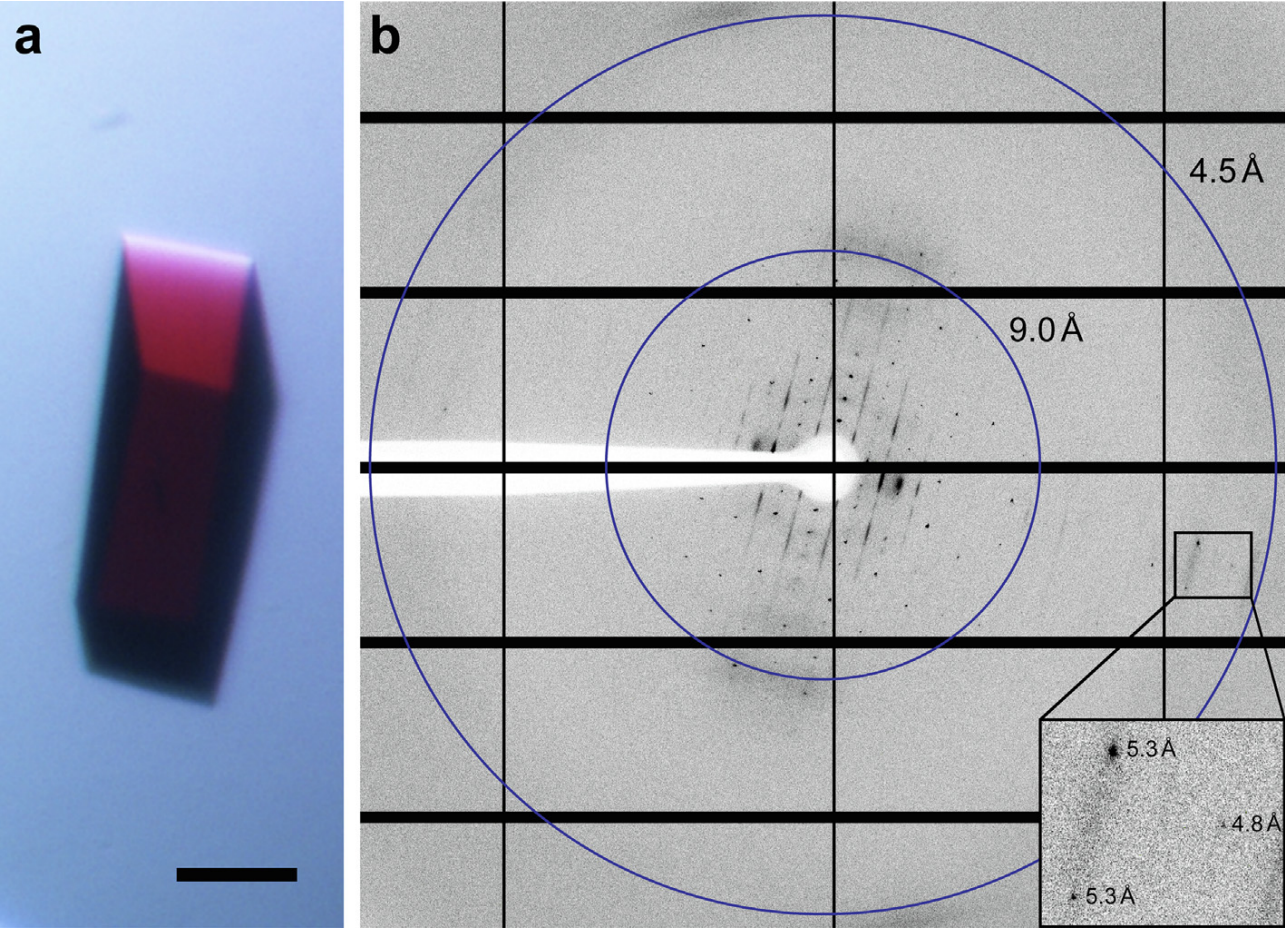
Representative three-dimensional GPR crystal and corresponding X-ray diffraction pattern. (a) Orthorhombic GPR crystal in the mother liquor exhibiting the intense red color associated with covalently linked retinal. Scale bar represents 50 μm. (b) X-ray diffraction pattern of GPR crystal recorded on an Eiger 16 M detector. Diffraction spots can be observed beyond 5 Å (see inset). Blue rings indicate resolutions of 9.0 and 4.5 Å, respectively. (For interpretation of the references to color in this figure legend, the reader is referred to the web version of this article.)

**Fig. 5:**
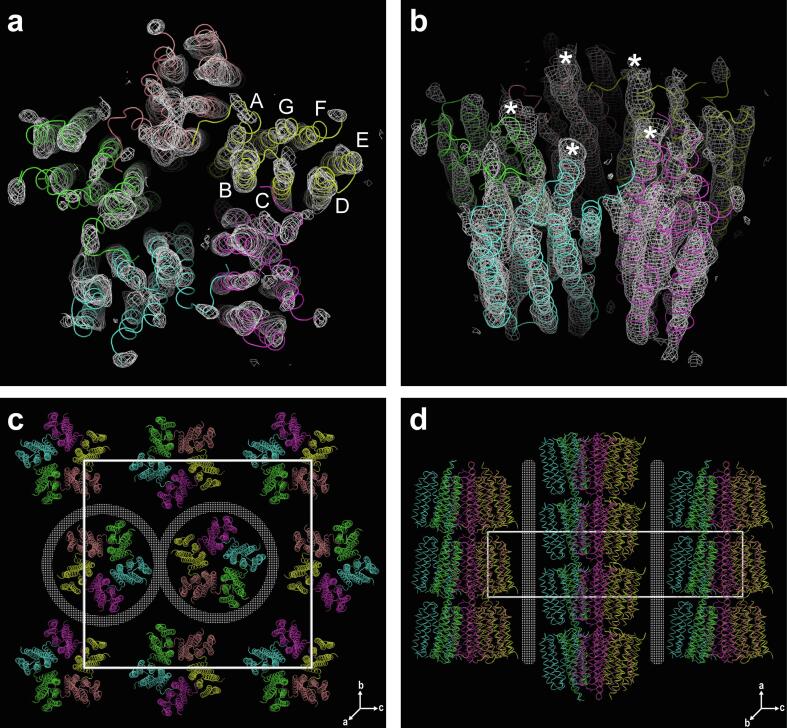
Electron density map and crystal packing in GPR crystals. Top view (a) and angled side view (b) of the electron density map contoured at 1.5 σ containing a C_α_ -backbone model of BPR (coloured by chain). The pentameric assembly of GPR is clearly visible and groups of elongated electron densities are discernable, corresponding to the seven transmembrane helices (labelled A-G) of the individual protomers. Potential crystal contacts between the BC-loops (loop connecting helices B and C) and the C-termini of adjacent pentamers are labelled with asterisks. Unit cell and crystal packing shown from top view (c) and side view (d). The unit cell with dimensions a = 53.42 Å, b = 190.29 Å and c = 208.73 Å is indicated as a white box in (c) and (d). Lattice directions are shown at the bottom right of corresponding panels. Lateral crystal contacts are mediated by the detergent micelles and are indicated by dotted areas. Pentamer backbone models are coloured by chain as in (a) and (b). The displayed structural information was prepared using PyMOL (https://pymol.org).

Three-dimensional protein crystals of oligomeric GPR were successfully grown under the described conditions (see Materials and methods). Red orthorhombic crystals (Fig. 4a) with lengths of 100–200 μm appeared after 2–4 days. GPR crystals were analyzed by X-ray crystallography and showed anisotropic diffraction to 4.44 Å resolution (Table 1 and Fig. 4b). The processed diffraction data were used to perform molecular replacement with Phaser (McCoy et al., 2007) using the pentameric *HOT75*BPR (PDB ID: 4KLY, ~80% amino acid sequence identity to GPR), the hexameric *Med12*BPR (PDB ID: 4JQ6, ~56% amino acid sequence identity to GPR) and the respective monomers of these BPRs as search models. A reasonable solution with Log Likelihood Gain (LLG) of 433 and translation function Z (TFZ) score of 12 (TFZ scores above 8 imply a successful solution) was found for the pentamer. On the other hand, the hexamer yielded an impossible solution (i.e., overlapping protomers of adjacent oligomers) and the monomers no solutions according to Phaser (McCoy et al., 2007). Despite the moderate resolution, the electron density was of sufficient quality to discern the overall crystal packing, the oligomeric assembly and even the transmembrane topology (Fig. 5). Importantly, the electron density unambiguously reveals that the purified protein is indeed a pentamer and not a hexamer (Fig. 5a and b), solidifying our previous assumptions. Groups of elongated electron densities are nicely discernible, corresponding to the seven transmembrane α -helices of GPR protomers (Fig. 5a, labelled A-G). Even a few loops, i.e., densities connecting the individual helices, are prominent. The crystal belongs to the *P*2_1_2_1_2_1_ space group and is built from alternating inverted columns consisting of pentamers forming head-to-tail contacts (Fig. 5c and d). The unit cell contains a total of four GPR pentamers, one per asymmetric unit. Crystal contacts are formed by the solvent accessible intra- and extracellular surfaces of the protein, potentially between the BC-loops (i.e., loop between helices B and C) and the C-termini (marked with asterisks in Fig. 5b) of adjacent pentamers. Lateral contacts seem to be mediated by the detergent micelles surrounding the pentamers (indicated by dotted areas in Fig. 5c and d).

**Table 1:** X-ray diffraction data statistics.

Beamline	X06SA, Swiss Light Source
Detector	Eiger 16M
Wavelength (Å)	1.0
Space group	*P*2_1_2_1_2_1_
Unit-cell parameters (Å; °)	a = 53.42, b = 190.29, c = 208.73; α = β = γ = 90
Anisotropic resolution limits^a^	
overall (Å)	4.85
along h axis (Å)	4.44
along k axis (Å)	5.61
along l axis (Å)	5.55
Resolution range (Å)^b^	47.57–4.44 (4.77–4.44)
R_meas_^c^	0.07 (4.00)
R_p.i.m._^d^	0.02 (1.10)
Measured reflections	85,824 (4,376)
Unique reflections	6,652 (333)
Mean *I/σI*	15.4 (0.9)
Completeness	
Spherical (%)	47.9 (12.7)
Ellipsoidal (%)^e^	87.7 (98.0)
Multiplicity	12.9 (13.1)
CC_1/2_^f^	1.0 (0.47)

a Anisotropic resolution limits were computed with AIMLESS (Evans and Murshudov, 2013) based on CC_1/2_ > 0.50.

b Statistics are for data that were truncated by STARANISO software (http://staraniso.globalphasing.org) to remove poorly measured reflections affected by anisotropy. Values in parentheses are for the highest resolution shell.

c *R*_meas_ as defined by Diederichs and Karplus (1997).

d *R*_p.i.m._ as defined by Weiss (2001).

e The completeness after the anisotropic correction was obtained by least-square fitting an ellipsoid to the reciprocal lattice points at the cut-off surface defined by a local mean *I/σI* threshold of 1.2, rejecting outliers in the fit due to spurious deviations, and calculating the fraction of observed data lying inside the ellipsoid.

f CC_1/2_ is the Pearson correlation coefficient of two-half data sets as described by Karplus and Diederichs (2012).

**Fig. 6:**
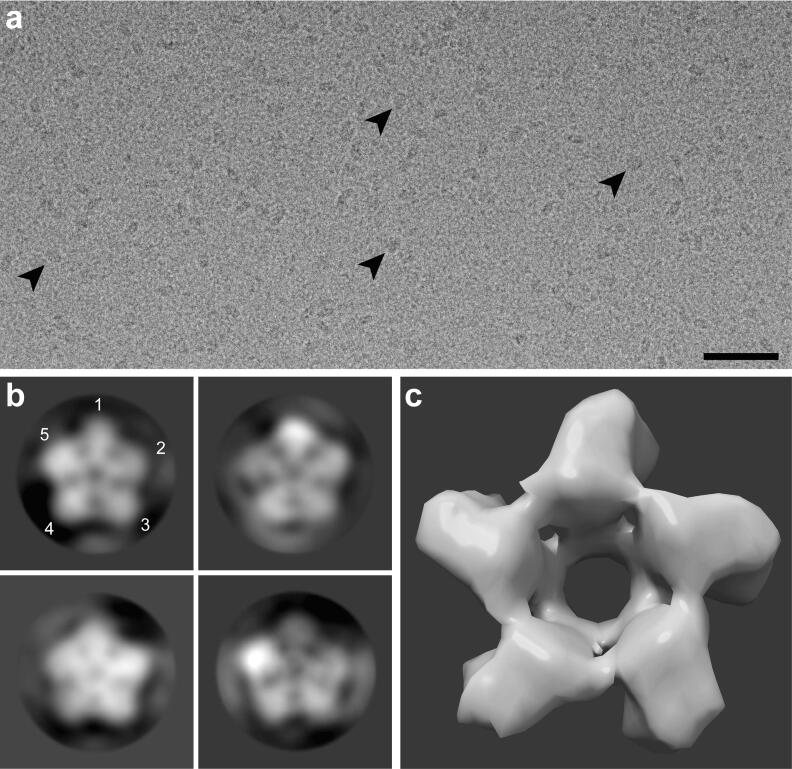
Single particle cryo-EM analysis of GPR. (a) Representative cryo-electron micrograph of purified GPR. Top view particles are indicated by black arrowheads. The scale bar represents 500 Å. (b) Selected 2D class averages of top views contain 1493 (top left), 5,656 (top right), 1748 (bottom left), and 1457 (bottom right) particles, respectively. The resolution of the classes is 14.6 Å (top left), 11.6 Å (top right), 12.2 Å (bottom left) and 11.6 Å (bottom right), respectively. The averages depict five distinct densities representing the pentameric assembly of GPR (protomers are numbered in the top left panel). The frame size is 220 Å, the mask diameter 175 Å and the particle diameter ~130 Å. (c) Simulated map of pentameric BPR (PDB ID: 4KLY) at a resolution of 15 Å calculated using Chimera (Pettersen et al., 2004). The frame size is 95 Å and the diameter of the map is ~90 Å.

The pentameric state of purified GPR, observed in 3D protein crystals, was confirmed in solution by single particle cryo-EM (Fig. 6). Fig. 6a shows a representative cryo-electron micrograph displaying differently oriented protein particles. To determine the oligomeric state of detergent-solubilized, purified GPR, top view particles (black arrowheads in Fig. 6a) were selected after initial automated particle picking and subjected to 2D classification. A selection of 2D class averages of top views is shown in Fig. 6b, displaying five distinct densities surrounding a central pore. A map of BPR (PDB ID: 4KLY) was calculated at 15 Å resolution for comparison (Fig. 6c). GPR class averages (Fig. 6b) have a diameter of ~130 Å compared to ~90 Å for the calculated BPR map (Fig. 6c).

## 3 Discussion

The presented purification procedure revealed the presence of two main GPR populations in solubilized membranes from untagged GPR expressing *E. coli* cells (Fig. 1a–c and Supplementary Fig. 1). The combination of IEC and SEC enabled the tag-free isolation of homogeneous and highly pure pentameric GPR (Fig. 1d and e). Compared to previous reports, we demonstrate here a procedure that yields a single oligomeric form (Fig. 1e) in contrast to a mixture of pentamers and hexamers (Hussain et al., 2015, Klyszejko et al., 2008, Maciejko et al., 2015, Shibata et al., 2018). Interestingly, the SEC elution volume of the putative GPR monomer was slightly different depending on which IEC peak (i.e., M or O) was analyzed (Fig. 1b and c). We hypothesize that the difference results from the more stringent wash and thus higher delipidation of the second IEC population (O) thus reducing the MW of the monomer. The stoichiometry of the oligomer was first estimated based on the MW of the protein-lipid-detergent complex, which was determined by SEC. The molecular simulations of the complexes using the estimated number of detergent molecules yielded convincing evidence for the pentameric assembly due to a lack of detergents for full coverage of the hydrophobic circumference in the hexamer (Fig. 3). Importantly, the assumed stoichiometry could be directly confirmed through analysis of three-dimensional protein crystals by X-ray crystallography. GPR crystals diffracted beyond 5 Å in one lattice direction (Table 1 and Fig. 4b). Such moderate resolutions preclude the building of atomic models, but allow the determination of oligomeric state and secondary structure elements. Phase information was gained by molecular replacement using the pentameric structure of the close homolog BPR. This yielded a reasonable solution with nicely defined electron density displaying the pentameric assembly of GPR and the heptahelical structure of the protomers (Fig. 5a and b). A detailed comparison with the structure of BPR is restricted due to the limited resolution of the electron density. However, the overall architecture of the GPR protomers reveals no significant differences compared to BPR. The lengths and orientations of the α -helices relative to the membrane plane in GPR are almost identical to BPR and no prominent bends or kinks are observed. The moderate and anisotropic nature of the diffraction data is reflected by two types of interactions in the crystal packing. The best resolution is found along the lattice direction (a-axis) where crystal contacts are formed by direct interactions between the hydrophilic surfaces of adjacent pentamers (Fig. 5d). Lateral contacts along the other two axes (b- and c-axes) seem to be only mediated by interactions between the detergent micelles surrounding the pentamers (Fig. 5c and d). These weak interactions explain the poor order of the crystal and diffraction along the respective axes. Detergent-mediated crystal packing has been observed previously and was likely the cause of poor diffraction quality (Palanivelu et al., 2006). It is interesting to note that the pentameric state of GPR is conserved in the closer of the two BPR homologs. *HOT75*BPR, with which GPR shares an amino acid sequence identity of ~80%, forms pentamers, whereas *Med21*BPR, with only ~56% identity, forms hexamers (Ran et al., 2013). To confirm the existence of pentameric GPR also in solution not only in crystals, the purified protein was analysed by single particle cryo-EM (Fig. 6). 2D class averages (Fig. 6b) of top view particles clearly display five well-defined densities corresponding to the protomers that are connected to a ring-like structure. The same features are also observed in the calculated 15 Å map of BPR (Fig. 6c). A smaller diameter is observed for the calculated map compared to the 2D class averages (~90 Å and ~130 Å), which is most likely due to the absence of a detergent micelle in the BPR model. The experimental evidence from single particle cryo-EM further corroborates our assignment of the pentameric state for native-like GPR purified with the presented procedure.

Proteorhodopsins, in particular the green-light absorbing proteorhodopsin, have been the focus of continuous research since their discovery 20 years ago. However, there has been controversy surrounding the oligomeric state of GPR, which is manifested by the various diverging reports (Edwards et al., 2014, Hoffmann et al., 2010, Hussain et al., 2015, Maciejko et al., 2015, Shibata et al., 2018, Stone et al., 2013). Different protein constructs and purification conditions have been used that, based on a variety of analytical methods, yield predominantly pentameric or hexameric GPR. However, it is notable that almost all preparations of wild-type GPR in mild detergents yield mostly pentamers (Hoffmann et al., 2010, Maciejko et al., 2015). In contrast, preparations commonly used for spectroscopic experiments, such as cysteine-less PR versions combined with the E108Q mutation and introduced cysteines for spin labelling, seem to yield mainly hexamers in mild detergents (Edwards et al., 2014, Hussain et al., 2015, Stone et al., 2013). All of the above mentioned GPR constructs were purified using a polyhistidine-tag. The addition of a purification tag may have unforeseen effects on the oligomerization or function of GPR. Tag-induced oligomerization is a phenomenon that has been known for a long time but is frequently neglected (Majorek et al., 2014, Mohanty and Wiener, 2004, Wu and Filutowicz, 1999). However, under the presented expression conditions using the described GPR construct, we found no evidence for His-tag-induced mixed pentamer/hexamer populations (Supplementary Fig. 2). Functional characterization yielded a consistent pK_a_ of about 7.4 for GPR with and without His-tag but revealed slightly blue-shifted absorption maxima at pH 6 and below in the presence of a His-tag (Fig. 2b). This coincides with the pH range in which histidine residues are predominantly protonated and suggests that the additional positive charge at the C-terminus affects the electronic environment of the retinal Schiff base. Based on these findings, experiments addressing the function of GPR might be affected by the presence of a His-tag at a pH below 6 and should be evaluated considering this observation.

We have provided here the first procedure for the selective, tag-free purification of pentameric GPR. The purified native-like protein is of high purity and homogeneity, and enabled structural analysis by single particle cryo-EM and X-ray crystallography of 3D protein crystals. Both methods provided direct and unambiguous evidence for the pentameric assembly of isolated GPR. Importantly, the presented purification procedure excludes the need for purification tags, which are commonly removed for downstream applications (Hirschi and Fotiadis, 2020). Furthermore, protein oligomericity can have severe effects on protease cleavage efficiency during protein purification and impede proper tag removal, thus resulting in significantly decreased yields and heterogeneous samples (Kenig et al., 2006). The homogenous and well-defined GPR sample in terms of oligomer stoichiometry provides an excellent starting point for future functional and structural studies.

## 4 Materials and methods

### 4.1 Cloning

The wild-type gene of GPR (GenBank: AY601905.1) without signal sequence was cloned into the previously described pZUDF21 vector (Ilgü et al., 2014), which was modified to contain a stop codon before the human rhinovirus 3C (HRV 3C) protease cleavage site and the decahistidine-tag (pZUDF21_stop_). Heterologous overexpression in *E. coli* yielded a tag-free recombinant protein lacking the C-terminal HRV 3C cleavage site and decahistidine-tag. The amino acid sequence corresponds to residues 19–250 and an N-terminal methionine. The His-tagged GPR version in pZUDF21 was cloned as described previously (Hirschi et al., 2019).

### 4.2 Overexpression of GPR in *E*. *coli*

The GPR constructs, i.e., untagged and His-tagged GPR, were overexpressed as described previously (Hirschi et al., 2019). Briefly, *E. coli* BL21(DE3) Rosetta2 cells were transformed with the GPR pZUDF21_stop_ (untagged) or GPR pZUDF21 (His-tagged) construct. The best expressing clone was selected for overexpression from small test expressions of individual bacterial colonies. Expression levels were estimated based on the red color intensity of the cell pellets. Cells were grown to an OD_600_ of about 0.3 at 37 °C and 180 rpm (Multitron, Infors HT). Then, the temperature was reduced to 18 °C and cell growth was continued. Expression was induced at an OD_600_ of about 0.75 with 0.1 mM isopropyl-β-D-thiogalactopyranoside (IPTG) and 5 µM all-*trans* retinal (dissolved in ethanol), followed by further incubation overnight. Cells were then harvested by centrifugation at 4 °C and 10,000×*g* for 5 min (Sorvall RC-5B centrifuge, DuPont Instruments), washed once with 50 mM Tris-HCl pH 8, 450 mM NaCl and stored at −20 °C until further use.

### 4.3 Tag-free purification of GPR

Frozen cells were thawed in a water bath and then lysed by five passes through a Microfluidizer (M-110P Microfluidizer, Microfluidics) at 1500 bar. Unlysed cells were removed by centrifugation (Sorvall RC-5B centrifuge, DuPont Instruments) at 4 °C and 10,000×*g* for 10 min. The supernatant containing the cell membranes was pelleted by ultracentrifugation (Optima L-90K ultracentrifuge, Beckman Coulter) at 4 °C and 150,000×*g* for 1 h. Pellets were homogenized and washed twice with 50 mM Tris-HCl pH 8, 450 mM NaCl by repeating the previous centrifugation. Finally, pellets were rinsed and homogenized in purification buffer (20 mM BTP-HCl pH 9.0, 50 mM NaCl). Membrane aliquots corresponding to one liter of bacterial culture each were flash frozen in liquid nitrogen and stored at −80 °C.

Membrane aliquots were solubilized in a total of 7 mL purification buffer containing 3% (w/v) 5-cyclohexyl-1-pentyl-β-D-maltoside (Cymal-5, Anatrace) at 4 °C on a turnover shaker overnight. The following steps were all performed at room temperature (RT). Solubilized membranes were ultracentrifuged (Optima L-90K ultracentrifuge, Beckman Coulter) at 100,000×*g* for 45 min. The supernatant was passed through a 5 mL Q Sepharose column (HiTrap Q FF, GE Healthcare) equilibrated with 20 mM BTP-HCl pH 9, 50 mM NaCl, 0.25% (w/v) Cymal-5. Bound proteins were eluted using a NaCl gradient from 50 to 500 mM (see Fig. 1a) on an ÄKTA purifier system (GE Healthcare) equipped with a UV–Vis detector (set to 280 nm and 520 nm) and an automated fraction collector. GPR containing fractions, i.e., fractions absorbing at 520 nm, were further purified by SEC on the same system using a Superdex 200 10/300 GL column equilibrated with 20 mM BTP-HCl pH 7.5, 150 mM NaCl, 0.25% (w/v) Cymal-5. Purity and homogeneity of purified GPR samples were analyzed by SDS-PAGE and BN-PAGE.

### 4.4 SDS-PAGE of purified GPR

Purified GPR samples were mixed with SDS loading buffer (80 mM Tris-HCl pH 6.8, 2% (w/v) SDS, 10% (v/v) glycerol, 0.0006% (w/v) bromophenol blue, 70 mM β-mercaptoethanol). A total of 2.5 μg GPR were loaded per lane and run on a hand-cast 16% SDS-polyacrylamide gel.

### 4.5 BN-PAGE of purified GPR

Purified GPR samples were mixed with BN-PAGE loading buffer (50 mM Bis-Tris-HCl pH 7.2, 50 mM NaCl, 10% (v/v) glycerol) supplemented with 0.5% (w/v) Cymal-5 and 0.375% (w/v) Coomassie Brilliant Blue G-250. A total of 2.5 μg GPR were loaded per lane on a precast 4–16% Bis-Tris NativePAGE gel (ThermoFisher). The gel was run using an anode buffer (50 mM Bis-Tris, 50 mM Tricine pH 6.8) and a dark blue cathode buffer (50 mM Bis-Tris, 50 mM Tricine pH 6.8, 0.02% (w/v) Coomassie Brilliant Blue G-250) for the first 30 min at 150 V, and a light blue cathode buffer (50 mM Bis-Tris, 50 mM Tricine pH 6.8, 0.002% (w/v) Coomassie Brilliant Blue G-250) for the following 80 min at 250 V. The gel tank was immersed in ice water during the whole run.

### 4.6 UV–Vis spectroscopic analysis of GPR

UV–Vis spectra of Cymal-5 solubilized, GPR containing *E. coli* membranes at different pHs were measured using a UV-1600PC spectrophotometer (VWR) in conjunction with the M.Wave Professional software (version 1.0.20). Samples were incubated with a final concentration of 100 mM potassium phosphate buffer at pH 4–10 with increments of 0.5 pH units. Spectra were recorded from 450 nm to 700 nm with 1 nm intervals. Values of absorption maxima were plotted against pH and fitted with an asymmetric sigmoidal function using GraphPad Prism software to determine apparent pK_a_ values.

### 4.7 Modelling of BPR-Cymal-5 micelle complexes

A protein-micelle complex of pentameric (PDB ID: 4KLY) and hexameric (PDB ID: 4JQ6) BPR in Cymal-5 were modelled with the Micelle Builder module of the CHARMM-GUI (Jo et al., 2008) (www.charmm-gui.org). Pentameric and hexameric BPR were inserted into a micelle system consisting of 155 and 103 Cymal-5 molecules, respectively. Boundaries of the membrane were defined according to the OPM (orientations of proteins in membranes) (https://opm.phar.umich.edu) database. The MW of the detergent micelle and thus the number of detergent molecules was estimated from SEC of GPR (see Results). Assuming a pentameric or hexameric state for purified GPR, the remaining mass can be attributed to either 155 or 103 Cymal-5 molecules. This represents an approximation of the maximal detergent content, since potentially co-purified lipids are neglected.

### 4.8 Crystallization of GPR

Purified GPR was concentrated to a final concentration of 8 mg/mL using a centrifugal filter with MW cut-off of 50 kDa (Amicon Ultra-4, Merck) and then ultracentrifuged (Optima MAX ultracentrifuge, Beckman Coulter) at 200,000 g for 15 min prior to crystallization. Crystallization trials were set up in 96-well 3-drop sitting-drop plates (SWISSCI) with a mosquito pipetting robot (TTP Labtech). Initial hits found using the commercial screens MemGold and MemGold2 (Molecular Dimensions) were further refined to the final condition comprising 50 mM MES pH 6, 25 mM MnCl_2_, 30% (v/v) PEG 400. Crystals appeared after 2–4 days. After another 2–4 days, crystals were harvested, flash frozen and stored in liquid nitrogen.

### 4.9 X-ray diffraction data collection and processing

X-ray diffraction data were collected at 100 K on an EIGER 16 M detector at the PXI (X06SA) beamline of the Swiss Light Source (SLS), Villigen, Switzerland. 1800 frames were recorded with an oscillation step of 0.2°, 0.1 s exposure time and at a filter transmission of 0.5 with a beam size of 80 × 40 μm (total dose of about 10.5 MGy). The crystal-to-detector distance was 470 mm and the wavelength 1.0 Å. Data were indexed and integrated using XDS (Kabsch, 2010) without truncation. Scaling and averaging were performed by the autoPROC (Vonrhein et al., 2011) package with truncation of the data at the best resolution along the h, k or l axis as determined by AIMLESS (Evans and Murshudov, 2013). Due to the anisotropic nature of the data, the STARANISO software (http://staraniso.globalphasing.org/cgi-bin/staraniso.cgi) was applied. Phaser (McCoy et al., 2007) of the PHENIX (Adams et al., 2010) software package was used for molecular replacement with the structures of pentameric *HOT75*BPR (PDB ID: 4KLY), hexameric *Med12*BPR (PDB ID: 4JQ6) and the respective monomers as search models.

### 4.10 Cryo-EM grid preparation, data collection and single particle analysis

2.5 µL of purified protein (3 mg/mL) were applied to a glow-discharged (10 mA, 120 s, 0.25 mBar) 300 mesh copper grid (Quantfoil R1.2/1.3) and vitrified using a Vitrobot™ Mark IV apparatus (Thermo Fisher Scientific) operated at approximately 100% humidity and 4–5 °C. Cryo-EM images were collected on a Tecnai F20 transmission electron microscope (Thermo Fisher Scientific) operated at 200 kV using a Falcon III direct electron detector in linear mode at a magnification of 80,000× (corresponding to a pixel size of 1.306 Å) and at a defocus range of −1.25 to −2.25 μm. Data were recorded in an automated fashion using the EPU software (Thermo Fisher Scientific). Movie stacks composed of 33 frames were recorded for 2.49 s with a dose of 2.27 e^−^/Å^2^/frame, resulting in a total accumulated dose on the specimen level of approximately 75 e^−^/Å^2^ per exposure. Dose-fractioned movies were subjected to beam-induced motion correction using MotionCor2 (Zheng et al., 2017) including dose-weighting. The contrast transfer function (CTF) parameters were estimated using ctffind 4.1.10 (Rohou and Grigorieff, 2015). Images of bad quality, e.g., with strong drift, resolution above 6 Å or high astigmatism, and ice-contaminated images were selected and discarded using the FOCUS software (Biyani et al., 2017). The template-free auto-picking procedure based on a Laplacian-of-Gaussian filter of RELION 3 (Scheres, 2012) was used to pick particles (total of 422,281 particles). After the first reference-free 2D class averaging with RELION 3 (Scheres, 2012) only top view class averages were selected and further separated from low quality particles by two rounds of 2D classification (total of 27,131 particles).

## Author contributions

S.H. and D.F. conceived and designed the experiments. S.H., D.K. and Z.U. performed the experiments. S.H., D.K. and D.F. analyzed the data. S.H. and D.F. wrote the manuscript. All authors contributed to manuscript revision and approved the final version.

## Declaration of Competing Interest

The authors declare that they have no known competing financial interests or personal relationships that could have appeared to influence the work reported in this paper.

## References

[b0005] Abramson J., Smirnova I., Kasho V., Verner G., Kaback H.R., Iwata S. (2003). Structure and mechanism of the lactose permease of *Escherichia coli*. Science.

[b0010] Adams P.D., Afonine P.V., Bunkóczi G., Chen V.B., Davis I.W., Echols N., Headd J.J., Hung L.-W., Kapral G.J., Grosse-Kunstleve R.W., McCoy A.J., Moriarty N.W., Oeffner R., Read R.J., Richardson D.C., Richardson J.S., Terwilliger T.C., Zwart P.H. (2010). PHENIX: a comprehensive Python-based system for macromolecular structure solution. Acta Cryst..

[b0015] Bamann C., Bamberg E., Wachtveitl J., Glaubitz C. (2014). Proteorhodopsin. Biochim. Biophys. Acta.

[b0020] Béjà O., Aravind L., Koonin E.V., Suzuki M.T., Hadd A., Nguyen L.P., Jovanovich S.B., Gates C.M., Feldman R.A., Spudich J.L., Spudich E.N., DeLong E.F. (2000). Bacterial rhodopsin: evidence for a new type of phototrophy in the sea. Science.

[b0025] Béjà O., Spudich E.N., Spudich J.L., Leclerc M., DeLong E.F. (2001). Proteorhodopsin phototrophy in the ocean. Nature.

[b0030] Biyani N., Righetto R.D., McLeod R., Caujolle-Bert D., Castano-Diez D., Goldie K.N., Stahlberg H. (2017). Focus: the interface between data collection and data processing in cryo-EM. J. Struct. Biol..

[b0035] Diederichs K., Karplus P.A. (1997). Improved R-factors for diffraction data analysis in macromolecular crystallography. Nat. Struct. Biol..

[b0040] Edwards D.T., Huber T., Hussain S., Stone K.M., Kinnebrew M., Kaminker I., Matalon E., Sherwin M.S., Goldfarb D., Han S. (2014). Determining the oligomeric structure of proteorhodopsin by Gd^3+^-based pulsed dipolar spectroscopy of multiple distances. Structure.

[b0045] Evans P.R., Murshudov G.N. (2013). How good are my data and what is the resolution?. Acta Cryst..

[b0050] Finkel O.M., Béjà O., Belkin S. (2013). Global abundance of microbial rhodopsins. ISME J..

[b0055] Friedrich T., Geibel S., Kalmbach R., Chizhov I., Ataka K., Heberle J., Engelhard M., Bamberg E. (2002). Proteorhodopsin is a light-driven proton pump with variable vectoriality. J. Mol. Biol..

[b0060] Gushchin I., Chervakov P., Kuzmichev P., Popov A.N., Round E., Borshchevskiy V., Ishchenko A., Petrovskaya L., Chupin V., Dolgikh D.A., Arseniev A.S., Kirpichnikov M., Gordeliy V. (2013). Structural insights into the proton pumping by unusual proteorhodopsin from nonmarine bacteria. Proc. Natl. Acad. Sci..

[b0065] Hempelmann F., Hölper S., Verhoefen M.-K., Woerner A.C., Köhler T., Fiedler S.-A., Pfleger N., Wachtveitl J., Glaubitz C. (2011). His75−Asp97 cluster in green proteorhodopsin. J. Am. Chem. Soc..

[b0075] Hirschi S., Fischer N., Kalbermatter D., Laskowski P.R., Ucurum Z., Müller D.J., Fotiadis D. (2019). Design and assembly of a chemically switchable and fluorescently traceable light-driven proton pump system for bionanotechnological applications. Sci. Rep..

[b0070] Hirschi S., Fotiadis D. (2020). Purification of membrane proteins by affinity chromatography with on-column protease cleavage. Methods Mol. Biol..

[b0080] Hoffmann J., Aslimovska L., Bamann C., Glaubitz C., Bamberg E., Brutschy B. (2010). Studying the stoichiometries of membrane proteins by mass spectrometry: microbial rhodopsins and a potassium ion channel. Phys. Chem. Chem. Phys..

[b0085] Hussain S., Kinnebrew M., Schonenbach N.S., Aye E., Han S. (2015). Functional consequences of the oligomeric assembly of proteorhodopsin. J. Mol. Biol..

[b0090] Ilgü H., Jeckelmann J.M., Gachet M.S., Boggavarapu R., Ucurum Z., Gertsch J., Fotiadis D. (2014). Variation of the detergent-binding capacity and phospholipid content of membrane proteins when purified in different detergents. Biophys. J..

[b0095] Inoue K., Ono H., Abe-Yoshizumi R., Yoshizawa S., Ito H., Kogure K., Kandori H. (2013). A light-driven sodium ion pump in marine bacteria. Nat. Commun..

[b0100] Jo S., Kim T., Iyer V.G., Im W. (2008). CHARMM-GUI: a web-based graphical user interface for CHARMM. J. Comput. Chem..

[b0105] Kabsch W. (2010). XDS. Acta Cryst..

[b0110] Karplus P.A., Diederichs K. (2012). Linking crystallographic model and data quality. Science.

[b0115] Kenig M., Peternel Š., Gaberc-Porekar V., Menart V. (2006). Influence of the protein oligomericity on final yield after affinity tag removal in purification of recombinant proteins. J. Chromatogr. A.

[b0120] Klyszejko A.L., Shastri S., Mari S.a., Grubmüller H., Müller D.J., Glaubitz C. (2008). Folding and assembly of proteorhodopsin. J. Mol. Biol..

[b0125] Maciejko J., Mehler M., Kaur J., Lieblein T., Morgner N., Ouari O., Tordo P., Becker-Baldus J., Glaubitz C. (2015). Visualizing specific cross-protomer interactions in the homo-oligomeric membrane protein proteorhodopsin by dynamic-nuclear-polarization-enhanced solid-state NMR. J. Am. Chem. Soc..

[b0130] Majorek K.A., Kuhn M.L., Chruszcz M., Anderson W.F., Minor W. (2014). Double trouble-buffer selection and His-tag presence may be responsible for nonreproducibility of biomedical experiments. Protein Sci..

[b0135] Man D., Wang W., Sabehi G., Aravind L., Post A.F., Massana R., Spudich E.N., Spudich J.L., Béjà O. (2003). Diversification and spectral tuning in marine proteorhodopsins. EMBO J..

[b0140] McCoy A.J., Grosse-Kunstleve R.W., Adams P.D., Winn M.D., Storoni L.C., Read R.J. (2007). Phaser crystallographic software. J. Appl. Cryst..

[b0145] Mohanty A.K., Wiener M.C. (2004). Membrane protein expression and production: effects of polyhistidine tag length and position. Protein Expr. Purif..

[b0150] Morizumi T., Ou W.-L., Van Eps N., Inoue K., Kandori H., Brown L.S., Ernst O.P. (2019). X-ray crystallographic structure and oligomerization of Gloeobacter rhodopsin. Sci. Rep..

[b0155] Ozaki Y., Kawashima T., Abe-Yoshizumi R., Kandori H. (2014). A color-determining amino acid residue of proteorhodopsin. Biochemistry.

[b0160] Palanivelu D.V., Kozono D.E., Engel A., Suda K., Lustig A., Agre P., Schirmer T. (2006). Co-axial association of recombinant eye lens aquaporin-0 observed in loosely packed 3D crystals. J. Mol. Biol..

[b0165] Pebay-Peyroula E., Garavito R.M., Rosenbusch J.P., Zulauf M., Timmins P.A. (1995). Detergent structure in tetragonal crystals of OmpF porin. Structure.

[b0170] Pettersen E.F., Goddard T.D., Huang C.C., Couch G.S., Greenblatt D.M., Meng E.C., Ferrin T.E. (2004). UCSF Chimera – a visualization system for exploratory research and analysis. J. Comput. Chem..

[b0175] Ran T., Ozorowski G., Gao Y., Sineshchekov O.a., Wang W., Spudich J.L., Luecke H. (2013). Cross-protomer interaction with the photoactive site in oligomeric proteorhodopsin complexes. Acta Cryst..

[b0180] Rohou A., Grigorieff N. (2015). CTFFIND4: Fast and accurate defocus estimation from electron micrographs. J. Struct. Biol..

[b0185] Schabert F.A., Henn C., Engel A. (1995). Native *Escherichia coli* OmpF porin surfaces probed by atomic force microscopy. Science.

[b0190] Scheres S.H.W. (2012). RELION: implementation of a Bayesian approach to cryo-EM structure determination. J. Struct. Biol..

[b0195] Shibata M., Inoue K., Ikeda K., Konno M., Singh M., Kataoka C., Abe-Yoshizumi R., Kandori H., Uchihashi T. (2018). Oligomeric states of microbial rhodopsins determined by high-speed atomic force microscopy and circular dichroic spectroscopy. Sci. Rep..

[b0200] Stone K.M., Voska J., Kinnebrew M., Pavlova A., Junk M.J.N., Han S. (2013). Structural insight into proteorhodopsin oligomers. Biophys. J..

[b0205] Váró G., Brown L.S., Lakatos M., Lanyi J.K. (2003). Characterization of the photochemical reaction cycle of proteorhodopsin. Biophys. J..

[b0210] Vonrhein C., Flensburg C., Keller P., Sharff A., Smart O., Paciorek W., Womack T., Bricogne G. (2011). Data processing and analysis with the autoPROC toolbox. Acta Cryst..

[b0215] Weiss M.S. (2001). Global indicators of X-ray data quality. J. Appl. Cryst..

[b0220] Wu J., Filutowicz M. (1999). Hexahistidine (His6)-tag dependent protein dimerization: a cautionary tale. Acta Biochim. Pol..

[b0225] Zheng S.Q., Palovcak E., Armache J.-P., Verba K.A., Cheng Y., Agard D.A. (2017). MotionCor2: anisotropic correction of beam-induced motion for improved cryo-electron microscopy. Nat. Methods.

