# Supplementary material for: Cryo-electron microscopic and X-ray crystallographic analysis of the light-driven proton pump proteorhodopsin reveals a pentameric assembly
Source: J Struct Biol X. 2020 Mar 8;4:100024. doi: 10.1016/j.yjsbx.2020.100024 (PMC7337067; doi:10.1016/j.yjsbx.2020.100024)
Supplement: Supplementary data 1 [file mmc1.docx]

**Supplementary Information**

**Cryo-electron microscopic and X-ray crystallographic analysis of the light-driven proton pump proteorhodopsin reveals a pentameric assembly**

Stephan Hirschi^a^, David Kalbermatter^a^, Zöhre Ucurum^a^, Dimitrios Fotiadis^a^

^a^ Institute of Biochemistry and Molecular Medicine, University of Bern, Bühlstrasse 28, 3012 Bern, Switzerland

**
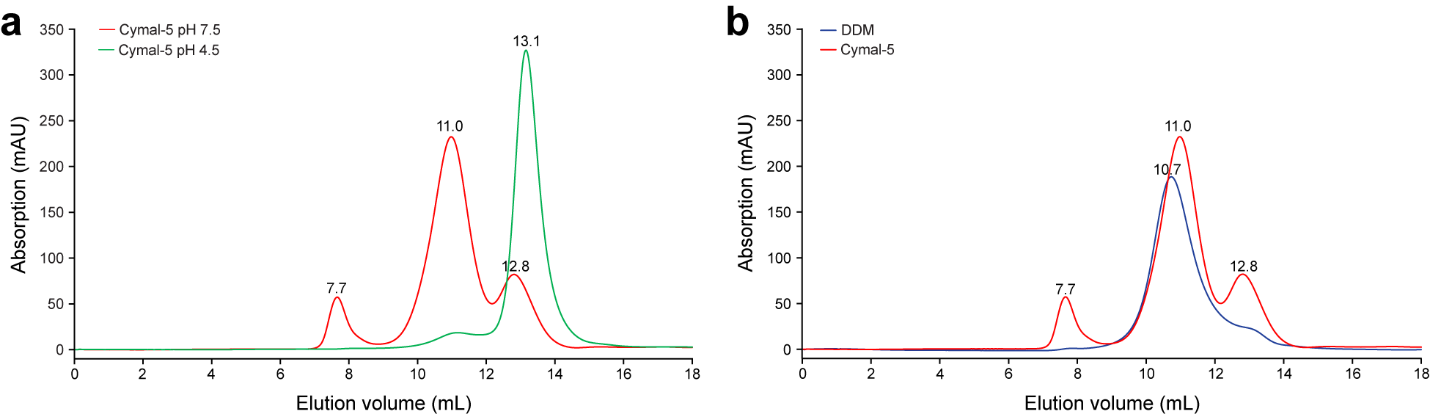
**

Supplementary Fig. 1: Size exclusion chromatography (SEC) analysis of detergent-solubilized membranes from *E. coli* cells expressing untagged GPR. (a) Membranes solubilized in 3% (w/v) Cymal-5 at pH 7.5 yield a small fraction of aggregates (7.7 mL), and a main oligomeric (11.0 mL) and a minor monomeric fraction (12.8 mL). When solubilized at pH 4.5, aggregates disappear and the majority of GPR oligomers dissociate into monomers. (b) Membranes solubilized in 2% (w/v) DDM yield GPR oligomers of comparable size to when solubilized in Cymal-5. The milder detergent DDM does not produce any aggregates and significantly less monomers compared to Cymal-5. GPR was detected using an UV-Vis detector set to 530 nm (SEC at pH 7.5) and 535 nm (SEC at pH 4.5).


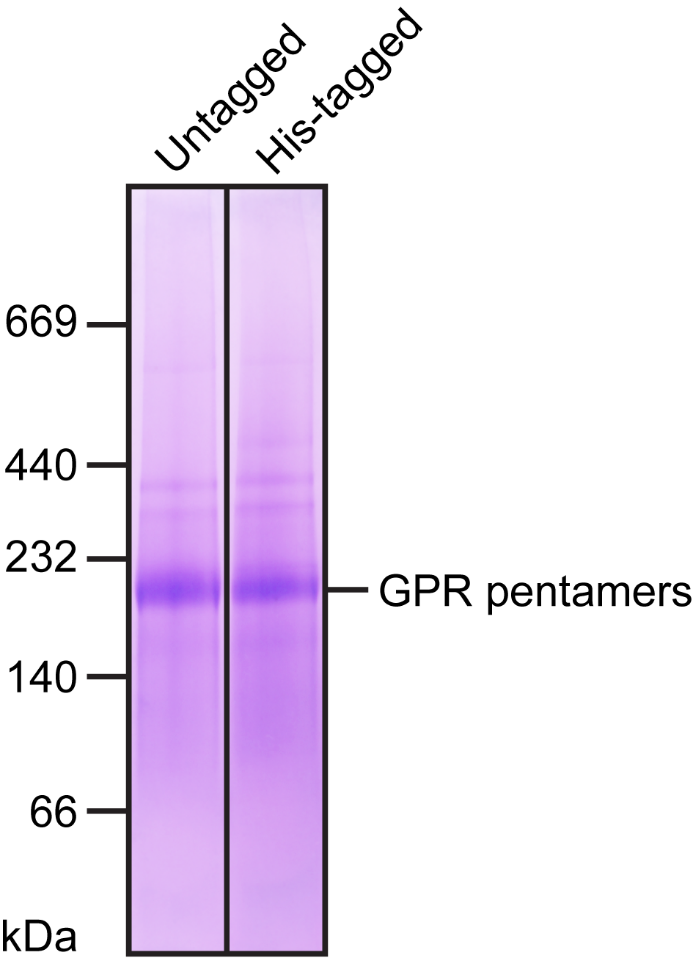


Supplementary Fig. 2: BN-PAGE of untagged and His-tagged GPR solubilized in DDM. Isolated *E. coli* membranes expressing GPR variants (untagged and His-tagged) were solubilized in 2% (w/v) DDM and analyzed on a precast 4–16% Bis-Tris NativePAGE gel after removing unsolubilized material by ultracentrifugation. In contrast to purified protein, all GPR populations present after expression are assessed by BN-PAGE of detergent-solubilized membranes. Both lanes look similar and GPR variants display a single prominent band at ~200 kDa, comparable to the purified GPR pentamers (Fig. 1e). This suggests that GPR, under the presented expression conditions, forms pentamers independent of the presence of a His-tag.
